# Potential Biochemical Mechanisms of Brain Injury in Diabetes Mellitus

**DOI:** 10.14336/AD.2019.0910

**Published:** 2020-07-23

**Authors:** Wei-Xing Ma, Jing Tang, Zhi-Wen Lei, Chun-Yan Li, Li-Qing Zhao, Chao Lin, Tao Sun, Zheng-Yi Li, Ying-Hui Jiang, Jun-Tao Jia, Cheng-Zhu Liang, Jun-Hong Liu, Liang-Jun Yan

**Affiliations:** ^1^Department of Pharmaceutical, University of North Texas Health Science Center, Fort Worth, Texas, USA; ^2^Chemical Engineering Institute, Qingdao University of Science and Technology, Qingdao, Shandong, China; ^3^Technological Center, Qingdao Customs, Qingdao, Shandong, China; ^4^Shantou University Medical College, Shantou, Guangdong, China

**Keywords:** diabetes, brain, cognitive impairment, Alzheimer’s disease, stroke

## Abstract

The goal of this review was to summarize current biochemical mechanisms of and risk factors for diabetic brain injury. We mainly summarized mechanisms published in the past three years and focused on diabetes induced cognitive impairment, diabetes-linked Alzheimer’s disease, and diabetic stroke. We think there is a need to conduct further studies with increased sample sizes and prolonged period of follow-ups to clarify the effect of DM on brain dysfunction. Additionally, we also think that enhancing experimental reproducibility using animal models in conjunction with application of advanced devices should be considered when new experiments are designed. It is expected that further investigation of the underlying mechanisms of diabetic cognitive impairment will provide novel insights into therapeutic approaches for ameliorating diabetes-associated injury in the brain.

## 1. Introduction

Diabetes mellitus (DM), including Type 1 and Type 2 DM, is a chronic disease that can damage many organs in the body, leading to symptoms such as hypertension, vision loss, cerebrovascular disease, cerebral edema and infarction. DM can also induce cortical and subcortical atrophy, white matter abnormalities, and perturbation of glymphatic function as well as overall dysregulation of cerebral metabolism [1-6]. Indeed, it has been established that DM-induced central nervous system (CNS) complications can cause cerebrovascular change and hippocampal synaptic plasticity alterations thereby contributing to reduced impulse condition velocity in presynaptic fibers and brain atrophy [7-9]. Additionally, several studies have also demonstrated that DM can induce Ca^2+^ dysregulation and accelerate brain aging that is closely related to Ca^2+^ dyshomeostasis [10]. Notably, it has been suggested that even if appropriate glucose control can limit the development of these severe complications, such limitation may not be sufficient to entirely prevent these complications from occurring [8].

The brain, as one of the most sophisticated organ in our body [11], plays an important role in glucose homeostasis by producing adaptive changes in energy intake, energy consumption, and hepatic glucose production [12]. Many cerebral-related injuries, disorders or dysfunctions can lead to irreparably serious consequences. Accumulating evidence indicates that more and more cerebral dysfunction is likely to be attributed to DM-related complications [7, 13-20], which might directly contribute to psychological and mental health outcomes [21] as well as other neurological deficits [22].

While stable glucose level is essential for cerebral metabolism and neuronal function and activity, prolonged periods of low or high glucose levels as well as rapid glucose fluctuations can result in neuronal injury [13]. Therefore, elucidating the mechanism of diabetic brain injury has been an increasing research interest. In this review, we attempted to summarize the current major biochemical mechanisms of diabetic brain injury, including hyperglycemia-induced cognitive impairment, diabetic Alzheimer’s disease (AD) and diabetic stroke. It should be noted that our review is not meant to exhaust all the possible mechanisms of diabetic brain injury documented in the literature.

## 2. Potential Mechanism in the Development of Diabetic Cognitive Impairment

### 2.1 Diabetes enhanced cerebral lesion deteriorates cognitive impairment

Increasing evidence indicated that DM is related to cognitive impairment, and the level of hyperglycemia and duration of DM have been shown to be linked with cognitive decline [23]. Cerebrovascular dysfunction is associated with cognitive impairment in patients with vascular dementia [24]. Abnormal variations in acetylcholine production, transport mechanism of choline, degradation of nicotinic and muscarinic receptors in the cerebral cortex can all result in cognitive impairment [25]. Recent analysis has revealed that gray matter atrophy and lesion of white material were related to declined cognitive performance that can be attributed to diabetes [14]. Cognition is particularly impaired when diabetes onset occurs during the midlife stage. Mid-aged adults with type 2 diabetes have exhibited alterations in neural function. In particular, reduced neural activation has been observed by functional imaging studies, indicating that cerebral changes probably precede diabetes onset in high risk individuals [26]. Another study based on advanced multimodal MRI showed that patients with impairment had a lower gray matter volume especially in the right temporal lobe and subcortical brain region. Concurrently, white matter volume and connectivity were decreased when compared to those without cognitive impairment [16]. Emerging evidence revealed by proton magnetic resonance spectroscopy also shows that adults with DM had a 9.3% lower ratio of N-acetylaspartate/creatine (NAA/cre) than in healthy subjects who were without DM. Thus, decreased NAA could be a potential biomarker of central neuronal dysfunction and may be useful in evaluating progression of neuropathy in DM [17]. Moreover, studies focusing on brain derived neurotrophic factor (BDNF), one of the most essential neurotrophic factors in the brain [27], and dipeptidyl peptidase-4 (DPP4), initially identified as a therapeutic target for type 2 diabetes [28], have demonstrated that both BDNF and DPP4 function as an antigenic enzyme involved in hyperglycemia, oxidative stress and inflammation-associated insulin resistance [29]). These studies provide evidence for the role of increased DPP4 and decreased BDNF in type 2-DM-related cognitive dysfunction [30], suggesting that DPP4 to BDNF ratio could be a novel biomarker for DM-related cognitive impairment [31].

### 2.2 Diabetes enhanced ca^2+^ dysregulation is a vital pathogenic mechanism of cognitive impairment

**Figure 1.**
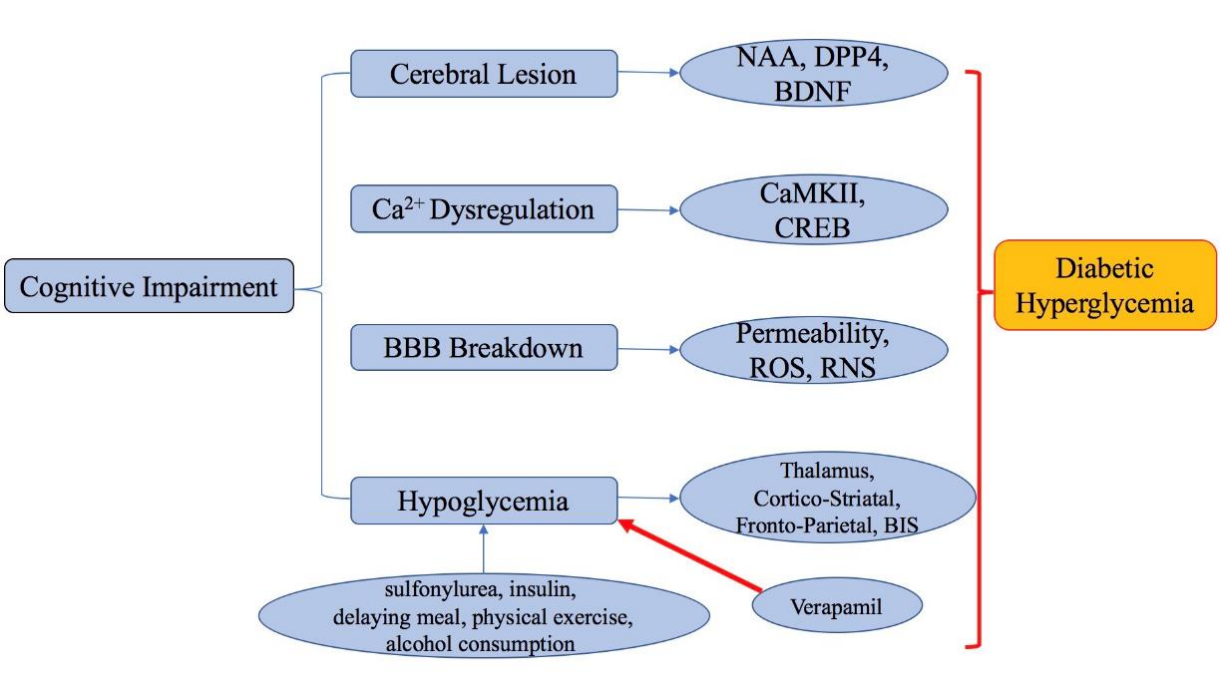
Signaling pathways and risk factors that are potentially involved in development of cognitive impairment induced by diabetes.

Tight regulation of calcium signaling is critical for many important functions of the brain, and subtle impairment in calcium signaling or disturbed calcium homeostasis can result in deleterious consequences including neuronal death [32] and age-related cognitive decline [33-35]. DM increases the risk of developing renal failure and contributes to impediment to phosphorus excretion from kidney, and thus leads to hyperphosphatemia. Under hyperphosphatemic condition, hypocalcemia is induced by interfering with phosphorus excretion. Moreover, phosphate can bind ionized calcium and remove calcium from the bloodstream [36]. As Ca^2+^ is indispensable for insulin and glucagon secretion, alterations in Ca^2+^ homeostasis caused by DM can thus impact blood glucose [37]. Many studies suggest that Ca^2+^ dysregulation could be a pivotal target of DM and might mediate the deleterious effects of DM on hippocampal function and behavior [10, 38]. Moreover, accumulation of intracellular free Ca^2+^ could also decrease the levels of Ca^2+^/calmodulin-dependent protein kinase II (CaMKII). As CaMKII regulated cAMP-response element binding protein (CREB) is involved in maintaining appropriate cognitive formation, disruption of calcium signaling by diabetic hyperglycemia can thus result in cognitive impairment [38] (Figure 1). Conversely, modulation of calcium-binding protein apoaequorin could have positive effects in treating cognitive dysfunction [39, 40]. Additionally, the transport of calcium across the inner mitochondrial membrane could also play a key role in neuronal physiology and pathology [41]. It has been reported that change in calcium signaling is likely involved in the compromise of mitochondrial ATP production [42] implicated in neurodegeneration [43]. Ca^2+^ signals in mitochondria stimulate ATP synthesis which is an important part of excitation-metabolic coupling. The communication between mitochondria and endoplasmic reticulum also plays an essential role in this metabolic fine tuning. And this communication can be impaired by DM leading to reduced cognitive functions [44]. Moreover, it has been demonstrated that a close relationship exists between mitochondrial dysfunction and cellular calcium homeostasis, which is likely contributed by metabolic increase in cytosolic free calcium and variations in intracellular calcium dynamics [45].

### 2.3 Diabetes enhanced blood-brain barrier (BBB) breakdown is possibly related to cognitive impairment

BBB forms a pivotal interface between the CNS and the general circulation [46] and contributes to CNS homeostasis [47]. Although mechanism underlying BBB dysfunction is missing [48], recent evidence indicates that BBB breakdown is prevalent, functionally relevant, and closely related to more rapid cognitive impairment [49]. Chronically increased oxidative stress, as one of the common pathological characteristics in DM, directly results in dysregulation of BBB tight junction proteins. This alteration, concurrent with DM hyperglycemia, may also increase BBB permeability [50] and result in further oxidative damage and glucose leakage from the blood to the brain parenchyma [51, 52]. DM-associated brain microvascular damage can also compromise BBB function, leading to cognitive impairment detected in neurodegenerative disorders [53]. Adaptive homeostatic responses at BBB might cope with increased oxygen and nutrient demand by regulation of influx and efflux via BBB transporters, which could change microvessel permeability, disrupts cerebral microenvironment and eventually leads to cognitive impairment [54]. Additionally, DM can trigger an increase in production of reactive oxygen species (ROS) and reactive nitrogen species (RNS) and a decrease in antioxidant enzyme activities, thereby accentuates brain damage [55] (Figure 1).

Over the past few decades, plenty of proteins belonging to the series of angioneurins have been considered as potential therapeutic targets for CNS caused by BBB dysfunction. A more recent review article states that certain angioneurins, including vascular endothelial growth factors, angiopoietins, platelet-derived growth factors and erythropoietin, are critical regulators of BBB integrity during brain injury and may vitally determine disease progression by multifaceted effects on the compromised BBB. These effects are based on the functional variety of angioneurins, which includes enhancing neuronal survival, stimulating the recruitment and proliferation of neural precursor cells, promoting neuronal plasticity, maintaining vascular integrity, and improving BBB tightness [56]. It has also been reported that activated brain mast cell, as the “first responder” in brain injury, may also contribute to CNS inflammation and cognitive dysfunction by promoting BBB disruption [57].

### 2.4 Diabetic hypoglycemia leads to cognitive impairment

Diabetic patients are exposed frequently to either hyperglycemia or hypoglycemia [58]. Hyperglycemia and hypoglycemia are two sides of a same coin. Hypoglycemia is a common consequence of diabetes treatment. There are several factors that could lead to hypoglycemia, including sulfonylurea therapy, insulin therapy, delaying or missing a meal, less physical activity, or increased alcohol consumption [59]. Exposure to hypoglycemia can cause a redistribution of cerebral blood flow towards the thalamus [60], and lead to brain damage and cognitive impairment [61-63]. Acute hypoglycemia decreases cerebral glucose consumption and might result in permanent brain injury. The areas of hypoglycemia-activated decrease in glucose consumption is more prominent in those of the brain where normal cerebral glucose consumption is higher [64]. However, hypoglycemia cannot be detected during general anesthesia, except for intermittent blood glucose monitoring or electroencephalogram readings. For diabetic patients, when the intraoperative bispectral index (BIS) value is abnormally low, hypoglycemia should be considered [64, 65]. One recent research concludes that, in patients (especially Type 1 DM) with hypoglycemia unawareness, there is a developing blunting of cerebral responses in cortico-striatal and fronto-parietal neurocircuits in response to mild-moderate hypo-glycemia. That’s why certain patients fail to respond to falling blood glucose levels [66]. In addition, another recent research states that calcium channel blocker, verapamil, could significantly decrease cerebral damage and cognitive impairment caused by severe hypoglycemia, which may be a useful approach to prevent hypoglycemia-induced cerebral injury [67]. It should be noted that while chronic hypoglycemia causes increased transportation, utilization and preservation of glucose in the brain, hyperglycemia-induced aggravation of ischemic brain injury is thought to be caused by increase in glucose concentration in the brain [68].

## 3. Potential DM-induced mechanisms leading to AD

AD, thought to be type 3 DM [69, 70], is the most frequent form of dementia and a growing public health burden. AD results in aggregation of amyloid β (Aβ) peptides in the brain, dysregulation of tau protein phosphorylation, insulin-resistance, lipid dysregulation, axonal loss, memory loss, and neuronal death [71-74]. The aforementioned BBB damage also could promote AD development [75]. It is well-established that DM increases the risk of AD and may play a causative role in the progress of AD pathogenesis [76].

Relevant studies suggest that the potential mechanisms of diabetes-induced AD might be due to dysregulation of glucose and insulin signaling in Type 2 DM. It is thought that insulin deficiency due to β cell dysfunction in full-blown Type 2 DM may cause AD by hampering insulin’s function in the brain [77-79]. Insulin presents critical functions in the CNS that goes far beyond glucose metabolism [80]. It is currently believed that aberrancy of glycogen synthase kinase-3 (GSK-3) could lead to AD [81], and GSK-3β might be the potential bridge between DM and AD. This is because GSK-3β abnormality could result in insulin resistance and insulin deficiency, thereby leading to phosphorylation dysregulation of tau protein (a pathological feature in AD) [82, 83].

It is also thought that inflammatory mechanisms can accelerate the core AD pathology development [84]. It has been reported that inflammation disrupts normal cerebral insulin signaling [85] and is caused by the accumulation of Aβ in the brain. Therefore, inflammation is considered a causative point in AD and is also thought to be an underlying link between AD and Type 2 DM [86]. Another factor receiving a lot of attention is toll-like receptors (TLRs), especially toll-like receptor 4 (TLR4). Aβ-mediated TLR4 activation deteriorates neuro-inflammation in AD [87]. Chronic TLR4 activation may result in insulin resistance in DM and Aβ deposition in AD, and may serve as a potential link between DM and AD [88]. Moreover, GSK-3 pathway, dysregulated by insulin resistance, enhances the Aβ plaque pathology and is closely related to tau misfolding and toxicity [89]. It should be noted that accumulation of Aβ modified by DM has been observed in wild type animals and animal models of AD, but not in humans [90]. The third important factor is nuclear factor-κB (NFκB). Hypothalamic inflammation activates the proinflammatory NFκB pathway, leading to glucose intolerance and insulin resistance. Moreover, within CNS, NFκB-driven hypothalamic inflammation employs a mechanism to induce the loss of blood pressure homeostasis, which is likely in parallel to producing glucose intolerance and insulin resistance [91]. TLRs aforementioned are primarily conveyed by the activation of NFκB which is a key early event in the pathobiology of DM and its complications. As a master regulator of brain inflammation, NFkB controls the expression of hundreds of genes implicated in innate immune responses, induces cytotoxicity, promotes cell division and apoptosis, and has a strategic role in the link between oxidative stress and inflammation [92]. Additionally, a recent research demonstrates that Type 2 DM with diabetic ketoacidosis (DKA) increases risk of AD [93]. Another recent research indicates that there is a correlation between apolipoprotein E polymorphism and the risk of AD, and ε3/4 and ε4 genotype of ApoE are the pathogenic factor for AD in patients with Type 2 DM [94]. Moreover, in terms of lipid regulation, ApoE is essential for lipid homeostasis, lipoprotein catabolism and cholesterol transport into cells. Under DM condition, there is an increased cholesterol flux in patients with AD [73]. It should also be pointed out that advanced glycation end-products (AGEs) are also involved in the complications of DM and are critical to the pathogenesis of AD. Patients suffering from AD combined with DM present an increase in cell damage via a receptor-for-AGEs-dependent mechanism, indicating that AGEs may enhance the vicious cycle of oxidative stress [95].

Therefore, it is clear that there are common pathophysiological mechanisms between DM and AD. Role of insulin and insulin resistance are the key nexus. When insulin receptor activity is hampered by DM, hypoglycemia could be induced which constrains glucose metabolism in the brain. That’s why diabetic patients are more at risk of deteriorating AD, and AD is called “Type 3 DM” [96, 97]. It should be pointed out that mechanisms underlying the alterations in macrovascular and microvascular cerebral blood flow (CBF) induced by DM should be further explored, because reduced CBF may initiate a series of events promoting cognitive impairment in AD [98, 99].

**Figure 2.**
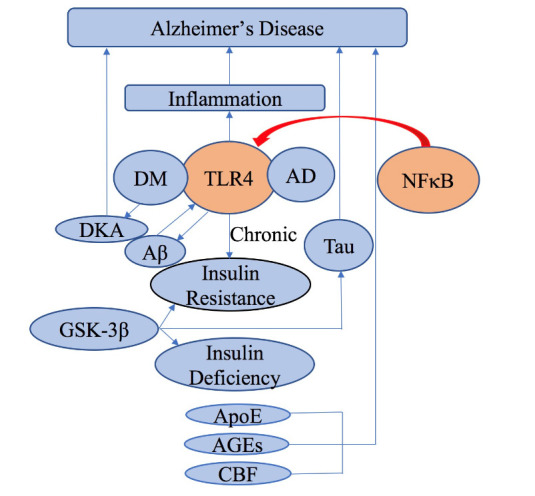
Signaling pathways and risk factors that are potentially involved in diabetic AD.

While many relevant studies have been conducted, the potential mechanisms linking DM to AD are still under debate [100]. We think it is necessary to carry out further studies with increased sample sizes and extended period of follow-ups to elucidate the effect of DM on cerebral damage in patients with AD [97]. Fig. 2 depicts all the potential mechanisms of diabetes-induced AD as described above.

## 4. Potential DM-induced mechanisms leading to Diabetic Stroke

DM is an increased risk factor for stroke; and about one fifth of patients with DM could die from stroke. In particular, DM duration has been shown to increase the risk of ischemic stroke (IS); with each additional year of DM duration, the risk for stroke is increased by 3% [101]. Stroke, especially IS, is currently the second leading cause of death and second cause of dementia after AD and often arises together with Type 2 DM [102, 103]. Mechanisms underlying DM may worsen consequence including vascular damage with stenosis of cerebral arteries leading to impairment of cerebral collateral flow [104]. Patients with DM presented an increased recurrence of stroke, especially IS, leading to neuronal cell death and infarction that accounts for about 87% of all strokes [63, 105, 106] when compared to non-DM patients [107, 108]. Patients suffering from acute ischemic stroke (AIS) with a history of prior stroke and DM could have increased serious cerebral injury [109]. A recent study focusing on the impact of DM on AIS also demonstrates that stroke recurrence was dramatically higher in DM patients than in prediabetic patients and healthy subjects (4.2% versus 0.7 versus 2.2%; P=0.005) [110]. And prediabetes, upon meta-analysis including big population, was also shown to be involved in increasing risk of new stroke in comparison to patients with IS or transient ischemic attack stroke (TIA) [111].

**Figure 3.**
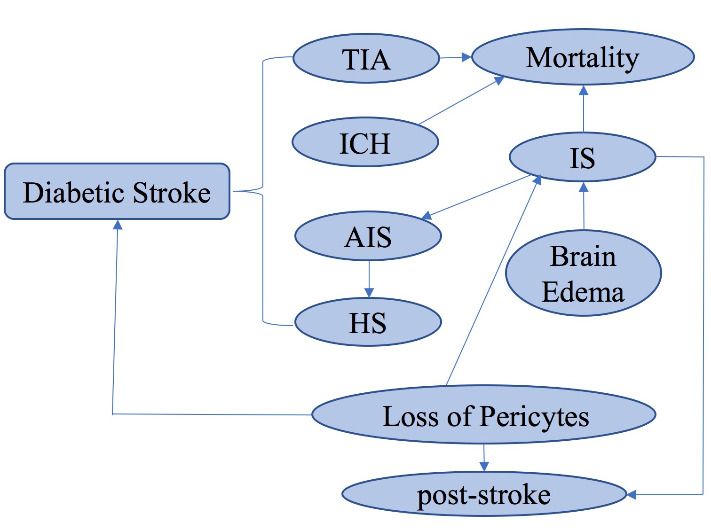
Signaling pathways and risk factors potentially involved in diabetic stroke.

Figure 3 presents the general pathways and risk factors involved in diabetic stroke discussed here. With DM being a comorbidity, patients with IS, TIA and intracerebral hemorrhage (ICH) had higher mortality [112]. The neurovascular unit (NVU) can be constrained by stroke, or hyperglycemia combined with DM, which serves as a common comorbidity in patients with AIS. Maintaining the NVU integrity and protecting NVU against damage are approaches to treating AIS. However, undesirable consequence such as AIS conversion to a worse hemorrhagic stroke (HS) is possible [113]. Thus, treatments for lowering glucose and decreasing vascular risk factors for stroke prevention should be urgently investigated [102]. Severity of loss of pericytes after an ischemic event may have important implications for stroke recovery but direct evidence is lacking. However, in terms of the crucial determent of pericyte density in vascular reparative response, it can only be speculated at this moment that extensive loss of pericytes (or pericytes injury) contributes to stroke and post-stroke cerebral vasoregression in diabetes. This is so as the relationship between pericyte loss or injury and stroke injury remains to be investigated. [18, 114]. It should be pointed out that post-stroke depression/anxiety is also a common neuropsychological consequence of IS [115]. In addition, brain edema is the primary cause of IS mortality. The mechanism underlying this phenomenon may be the rapid disturbance of water contributing to abnormal elevation in intracranial pressure and/or intracranial fluid [116, 117].

Therefore, while antidiabetic medicines for stroke in patients with DM are vigrously being developed [118], further studies should also concentrate on potential mechanism of DM-induced different phase of stroke [119]. This might be helpful in identifying specific pathogenic mechanisms underlying cerebral damage caused by DM potentiated stroke. However, most of animal model studies focusing on stroke research need animal sacrifice, which limits the ensuing cognition or behavioral studies on the same animal. Additionally, reproducibility may also be an issue. It seems that the novel approach using magnetic resonance imaging for stroke evaluation may enhance reproducibility [120].

### Summary

In this review, we have summarized current biochemical mechanisms of diabetic brain injury based on publications published mainly between 2017 and 2019, which accounts for more than two-thirds of all the citations. While many published articles elucidated similar mechanisms in different perspectives, conditions, experiments, hypotheses and explanations, certain controversies remained common among these articles. Further investigations combining better-designed animal models with advanced and high-precision devices are badly needed to enhance reproducibility of experiments conducted by disparate investigators.
